# The effect of anti-nerve growth factor monoclonal antibodies on the clinical signs of degenerative joint disease in cats

**DOI:** 10.18849/ve.v8i4.623

**Published:** 2023-10-18

**Authors:** Lydia Seeley+

**Keywords:** FELINISED MONOCLONAL ANTIBODIES, OSTEOARTHRITIS, PAIN MANAGEMENT, CATS, FELINE

## Abstract

**PICO question:**

Are felinised anti-nerve growth factor monoclonal antibodies (frunevetmab) effective at reducing the clinical signs of pain and immobility in cats with degenerative joint disease when compared with no treatment?

**Clinical bottom line:**

**Category of research:**

Treatment.

**Number and type of study designs reviewed:**

Three peer-reviewed randomised controlled trial treatment studies, two of which were pilot studies.

**Strength of evidence:**

Moderate.

**Outcomes reported:**

All three studies concluded that there was a statistical reduction in pain and an improvement in mobility in the groups administered frunevetmab, when compared to the groups administered the placebo.

**Conclusion:**

There is moderate evidence suggesting that the administration of frunevetmab by injection led to a reduction in pain and an increase in mobility. Injections were given at day 0; day 0 and 28, or day 0, 28 and 56 depending on the study. Further research should be conducted to ensure repeatability, involving more objectively measured outcomes to reduce the reliance on subjective measures which are more likely to have associated bias.

## How to apply this evidence in practice

The application of evidence into practice should take into account multiple factors, not limited to: individual clinical expertise, patient’s circumstances and owners’ values, country, location or clinic where you work, the individual case in front of you, the availability of therapies and resources.

Knowledge Summaries are a resource to help reinforce or inform decision making. They do not override the responsibility or judgement of the practitioner to do what is best for the animal in their care.

## The evidence

After exclusion criteria were applied, three studies were appraised in this Knowledge Summary; all three studies were randomised double-blinded controlled clinical trials conforming to CONSORT guidelines, providing an overall moderate evidence-base.

All of the studies used subjectively-measured questionnaires completed by the cat owners, which meant there was potential for bias. The studies were double-blinded to mitigate this bias. All three studies used the same measurement tools, Feline Musculoskeletal Pain Index (FMPI), and Client-specific Outcome Measures (CSOM), and veterinary orthopaedic examination, which made them comparable, and despite the strong placebo-effect, a positive statistical difference was demonstrated in all three studies. Both pilot studies (Gruen et al., 2016; and Gruen et al., 2021a) also implemented the use of objective measurement by accelerometers (activity-trackers), however, objective measurement was not used in the final study (Gruen et al., 2021b).

Of the subjective measurements, the CSOM is yet to be validated, although it has been used in other treatment studies (Lascelles et al., 2007). The same applies for the FMPI, but the FMPI is useful for segregating pain-free cats from those suffering from osteoarthritis (OA) (Benito et al., 2013).

Furthermore, all three studies were carried out by the same author and research group. There is a declared conflict of interest that all the studies were supported by a large pharmaceutical company (Zoetis) with active study involvement of company employees, which creates an innate risk of sponsorship and single group bias. However, all three papers were available with ‘open access’ which shows transparency by the company.

All three studies compared injectable frunevetmab with placebo (saline injection), however different doses were used of the drug, meaning the studies were not directly comparable. Despite the variation in dose, similar impacts on lameness were reported. There were positive statistical differences across all three studies, therefore a conclusion was reached that injectable felinised anti-nerve growth factors (NGF) monoclonal antibodies improve clinical signs by providing analgesia and improve mobility in cats with degenerative joint disease when compared to a placebo.

## Summary of the evidence

**Gruen et al. (2016):** 

Population:	Exclusively indoor cats. Over the age of 1-year-old. Radiographically-evident degenerative joint disease associated pain and mobility impairment in at least two joints.
Sample size:	34 cats.
Intervention details:	Cats were randomly allocated into three groups and administered a single treatment of NV-02 (now frunevetmab) or placebo (saline) subcutaneously: Group 1 (n = 11) – 0.4 mg/kg NV-02. Group 2 (n = 12) – 0.8 mg/kg NV-02. Group 3 (n = 11) – 0.21 ml/kg or 0.42 ml/kg placebo. All personnel were blinded except for the pharmacy staff who were responsible for dispensing the treatment.
Study design:	Pilot study: randomised, placebo-controlled, double blinded, clinical trial.
Outcome studied:	Baseline data was established in the 14 days prior to the first treatment being administered. Then average weekly activity and debility scores were assigned by the owner over a period of 11 weeks. An orthopaedic examination was carried out by a veterinarian, and the joints were given a pain score before being radiographed for inclusion in the study. Objectively assessed: Measurement of the cats’ minute-by-minute activity by an accelerometer worn on a collar or harness around the cat’s neck, which was averaged to generate a single figure of average weekly activity. Subjective assessment: Questionnaires were completed by the primary owner of each cat at 3-weekly intervals during the 11-week study period (weeks 2, 5, 8 and 11). Client-specific outcome measure (CSOM) – clients were asked to identify three activities that their cat struggled to perform which would be used to construct CSOM assessment. They rated their cat’s ability to perform these tasks on a Likert scale. The scores for each activity were combined. Feline musculoskeletal pain index (FMPI) – the owners assigned scores on a Likert scale about the ability of their cat to perform 17 activities. They then had to mark their cat’s level of pain on a 100 mm visual analogue scale. Owner assessment of whether active treatment had been administered – on day 77, owners were asked whether they thought their cat had been administered frunevetmab or placebo.
Main Findings (relevant to PICO question)	No significant differences were found between the two treatment groups, so they were combined for analysis. Significant increase in measured activity levels recorded by the activity monitors after 4, 5, 6, 7 and 8 weeks in the combined treatment groups when compared to the placebo group. The scores of the CSOM questionnaires were significantly improved 3 weeks after administration when compared to the placebo (P = 0.035). There was no significant improvement in CSOM scores at the other time points during the study. At day 35 (3 weeks after treatment) the improvement in the treatment groups’ CSOM score equated to 5.5 (55% decrease in disability and pain) (where >50% is considered successful in human medicine), compared to 22% (difference of 2 on CSOM score) in the placebo group. No significant differences in scores between the groups at day 56 and 77 (P = 0.466 and P = 0.673 respectively). There was an improvement in FMPI scores in both the treatment and placebo groups, but no significant difference between the two groups at any time throughout the study (P = 0.061 at day 35, P = 0.127 at day 56, and P = 0.456 at day 77). 83% of owners were able to correctly identify that their cat had received the treatment, versus 45% of owners correctly identifying that their cat had been administered the placebo. These data showed a positive analgesic effect of this monoclonal antibody treatment. Allergic response was not noted in any cat, six adverse events were recorded in the treatment groups. Total protein and serum globulin concentrations were significantly higher at day 77 in the treatment groups, however, change within groups was not significant over time for any variable.
Limitations:	Pilot study of a novel treatment, therefore power analysis could not be carried out, a limited population size was used (n = 34). Only indoor cats were used, so the group was not representative of the whole cat population. There was a significant caregiver placebo effect due to the subjectivity of the owner-based CSOM and owner assessment questionnaires. The placebo effect was to a greater effect mitigated by randomisation. Use of accelerometers, increased activity does not confer improvement in all cases. For example, higher readings could be obtained in cats irritated by the collar, those making more frequent trips to water or toilet facilities, or due to other environmental factors.

**Gruen et al. (2021a):** 

Population:	Generally healthy cats, and cats with stable medical conditions (including stage I or II IRIS renal disease) determined by appropriate investigation were included in the study. Exclusively indoor cats. All breeds, both sexes and neuter status. Over the age of 6 months. Client-perceived clinical signs and radiographically-diagnosed degenerative joint disease (DJD), and pain in at least two joints at veterinary orthopaedic assessment. Also score of >7 on CSOM questionnaire. The sample population had a mean age of 12–13 years old. Cats were excluded if on medication (except joint supplements and diets if they had received them for at least 45 days prior to the start of the study), they had significant comorbidities, pregnant or lactating, or having / had major surgery during / 1 month prior to the study period. Multisite field study (15 small animal clinics in the US).
Sample size:	126 cats.
Intervention details:	Cats were screened at their centre by a designated veterinarian at least 8 days prior to entry into the study, with a full clinical exam (including standardised neurological and orthopaedic examination), complete blood count, serum biochemistry, urinalysis, and radiographs on the affected joints being carried out. The cats were randomly allocated into three groups based on order of entry into the study. Each cat received two injections 28 days apart: Group 1 (n = 42) – frunevetmab intravenously on day 0, and subcutaneously on day 28 (dose of 7 mg or 14 mg which results in a range of 1.0–8 mg/kg depending on bodyweight). Group 2 (n = 43) – frunevetmab subcutaneously on day 0 and day 28 (dose of 7 mg or 14 mg that resulted in a dose range of 1.0–9 mg/kg depending on bodyweight). Group 3 (n = 41) – equivalent volume of placebo intravenously on day 0, and subcutaneously on day 28. All of the personnel involved were blinded to the treatment administered, except the treatment administrator. The investigating veterinarian could delegate the physical assessment to another veterinarian.
Study design:	Pilot field study: prospective randomised, placebo-controlled, double-masked clinical trial (multi-site).
Outcome studied:	Baseline data was gathered for a minimum of 8 days prior to the start of the study. Objectively assessed: The activity levels measured continuously throughout the study by an activity monitor attached to each cat’s collar. After 56 days the data was downloaded and a weekly average of per minute activity compared to the baseline was calculated, and then compared between the three groups. Veterinary orthopaedic examinations were carried out at screening, days 28 and 56 by the same veterinarian each time. Total pain and total disability scored were recorded. Safety outcome measures based on physical and neurological examinations (days 28 and 56), injection site (days 0, 28, and 56), clinical pathology (screening and day 56) and owner reported adverse events (anytime). Subjectively assessed: Questionnaires completed by the primary owner of each cat 8 days prior to the study and then every 2 weeks at days 0, 14, 28, 42 and 56; Client-specific outcome measures questionnaire (CSOM) – the ability of the cat to perform three activities tailored to the individual cat (selected prior to day 0) on a 1–5 scale. A reduction of ≥2 from the day zero score was defined as a treatment success. Feline Musculoskeletal Pain Index (FMPI) – owner rated their cat’s ability to perform 14 activities on a Likert scale. They were also required to assign the cat’s pain a score, rate their quality of life, and happiness. A reduction in the score of ≥10 was defined as a treatment success. Owner global assessment – on days 28 and 56, the owners were asked to assess the treatment’s success in controlling the clinical signs of DJD in their cat (excellent, good, fair, poor). Treatment success was defined as those who rated good or excellent, meaning an improvement of at least 50%. The number of treatment successes in each group were compared on days 14, 28, 42, and 56 for all three scales. Owner reassessments were performed every 14 days from the start of the study. The same examining veterinarian carried out physical and orthopaedic examinations on the cats in each clinic on days 0, 28 and 56. On day 56 (± 3), the cats returned to the clinic for a final physical and orthopaedic exam.
Main Findings (relevant to PICO question)	The two frunevetmab-treated groups showed no statistical difference between them in any section of the study, so were combined for comparison with the placebo. All the cats in the study had a decrease in their weekly activity measured by accelerometry when compared to the baseline. At the specified cut-off point, there were significantly higher success rates among the frunevetmab-treated group at weeks 1 (77% vs 58%), 2 (80% vs 51%), 3 (69% vs 41%), 5 (60% vs 32%) and 7 (55% vs 33%). The author suspected a falsely elevated baseline due to an increase in activity as a result of having to wear an unfamiliar collar. Significantly more cats, 54/71 (76.1%), were considered treatment successes based on the CSOM at days 42, and 56 (57/71 (80.3%)) when compared to the placebo, but not on days 14 and 28. There was a statistical difference in the owner’s global assessments in the frunevetmab-treated groups compared to the placebo-treated group on both day 28 (41/74, 55.4% vs. 10/38 (26.3%)) (P = 0.0134) and 56 (51/70 (72.9%) compared with 12/37 (32.4%)) (P = 0.0030). The FMPI found significantly more cats had a treatment success on day 42 (P = 0.0076) and day 56 (P = 0.024) for questions 1–17 of the FMPI which asked about the amount of pain, quality of life and ability to perform specified activities. No statistical differences were found for questions regarding ease of movement when compared to the placebo. There were significantly better responses for the treatment group than the placebo group on day 56 and on day 42 for questions concerning jumping up and down. There was no significant difference in total pain score found by veterinary orthopaedic exam, or total debility score in either the treatment or placebo group when days 28 and 56 were compared. Six cats were withdrawn from the study, three from dermatological effects associated with the collar around the neck, and three from other adverse effects (including perceived lack of efficacy).
Limitations:	Pilot study with a good sample size (n = 126), power calculations were not performed. Only indoor cats were used. Owner-based questionnaires carry some bias due to subjectivity and placebo-effect. Variable dose ranges of the drug were administered. Measuring activity levels may not be the best way to assess pain caused by DJD in cats, ability to perform activities may be a better measure. Multisite study, not the same veterinarian carrying out physical and orthopaedic examination throughout the study. Veterinary assessments of chronic pain are not representative as the cats are stressed in an unfamiliar environment in the clinic. Cats went home between injections so other factors affecting mobility could not be controlled. The study was only carried out for 56 days, which is a relatively short period of time.

**Gruen et al. (2021b):** 

Population:	Owned cats over the age of 6 months and >2.5 kg with owner noted clinical signs of osteoarthritis (OA). Any breed and gender. Naturally occurring OA in two or more joints with associated pain and reduction in mobility, with a minimum Client Specific Outcome Measures (CSOM) score of 7. Radiographs were carried out to diagnose OA and establish eligibility, unless previously performed within 12 months. Cats with stable chronic medical conditions (for example Chronic Kidney Disease (CKD) Internation Renal Interest Society (IRIS) score i-ii) were included. Cats were not permitted to have had any treatment with monoclonal antibodies prior to the study, and they were also not permitted to be on any other medications during the study, with the exception of oral nutraceuticals if they had been administered these at least 60 days and 45 days respectively prior to day 0. Cats were excluded where pregnant / lactating, had undergone major surgery within 1 month, had neurological conditions, or had planned elective surgery during the study period. Multi-site study involving 21 veterinary clinics in the USA.
Sample size:	275 cats.
Intervention details:	Baseline screening was carried out 3–14 days prior to the study; Complete blood count, serum chemistry urinalysis, full neurological, physical and orthopaedic examinations, radiographs, and owner Clinical Metrology Instruments (CMIs). Cats were randomly assigned to a group by an electronic data system on entry to the study, a superior randomisation system. Each cat received a single injection on days 0, 28 and 56: Group 1 (n = 182) – subcutaneous injection 1.0 mg/kg frunevetmab. Group 2 (n = 93) – placebo (saline) subcutaneous injection of the same volume. The treatment and placebo were assigned different codes so that the study was completely blinded except to the dispenser at each practice. The codes were not broken until the statistical analysis. On days 7 and 112, the owners received a telephone call to check on their cat’s status.
Study design:	Prospective, randomised, placebo-controlled, parallel-group, double-blinded, superiority study.
Outcome studied:	Baseline measurements were established at screening 3–14 days before the start of the study, and on day 0. Subjective assessment: Client-specific Outcome Measures (CSOM) were carried out at days 0, 28, 56 and 84, and at the time of each treatment being administered. Owner global assessment of the treatment’s success at reducing the clinical signs (excellent, good, fair, poor) on days 28, 56 and 84. Veterinary physical and orthopaedic evaluations assigning a score to the level of effusion, crepitus, and thickening, were carried out on days 28, 56 and 84 performed by the same veterinarian at each clinic for consistency. Safety outcome measures.
Main Findings (relevant to PICO question)	13 were removed from the study prior to day 56. Eight were censored from treatment success but not safety, and seven were fully censored. A significantly higher percentage of cats in the treatment group (118/178 [66.7%]) achieved treatment success based on a reduction in the CSOM score by ≥2 when compared to the placebo group (48/93 [52.06%]) on day 28 (P = 0.02). At day 56 the treatment group also had a significantly higher treatment success rate (133/176 [75.91%]) than the placebo group (58/91 [64.65%]) (P = 0.031). At day 84 there was a higher success rate among the treatment group (127/167 [76.47%]) than the placebo group (60/89 [68.09%]), but this was not significant (P = 0.08). Across all treatment time points, total CSOM scores were significantly lower in the treatment group compared to the placebo group. Owner global assessment was significantly higher in the treatment group on days 28 (P = 0.03) and 56 (P = 0.04) but not for day 84. Veterinary orthopaedic assessment gave significantly lower pain scores in the frunevetmab group on days 56 (P = 0.04) and 84 (P = 0.04) but not on day 28 (P = 0.3). A variety of adverse events were reported, but the majority deemed as unlikely related to treatment apart from skin disorders which occurred more in frunevetmab treated cats (32/182) vs placebo (8/93).
Limitations:	The research was funded by a pharmaceutical company and carried out by its employees, making sponsorship bias possible. Different veterinarian performing the orthopaedic exam in each clinic, so there was a lack of consistency across the whole study. High level of placebo-effect which got stronger as the study went on likely due to expectation bias due to knowledge of pilot trials, or due to the longer trial length and larger sample size. The study was carried out over a short period, long-term effects were not observed.

## Appraisal, application and reflection

There is evidence that the long-term use of non-steroidal anti-inflammatory drugs (NSAIDs) in the treatment of osteoarthritis (OA) is beneficial in cats (Gunew et al., 2008). There are, however, concerns regarding the long-term use of NSAIDs, including adverse drug reactions, and particularly around the difficulty in administering oral medication, especially when the palatability is low, and when the cat does not live exclusively indoors, or grazes throughout the day (Sivén et al., 2017).

Overall, the three clinical trials in the studies appraised in this Knowledge Summary were well-designed and carried a moderate level of evidence. The biggest limitation of all three studies was the sample size, typical for veterinary studies. Further studies should be carried out with larger sample sizes across an increased number of clinical settings. Another limitation of the studies is that they were all carried out by the same research group, and had received funding from a pharmaceutical company. A conflict of interest was declared. Ideally, further research would be carried out by another research group to reduce the possibility that there was a level of potential sponsorship and single group bias.

The study design and the implementation of clinical trials was well executed. The cats were enrolled onto each study and then randomly allocated to either a treatment or placebo group. In the first pilot study (Gruen et al., 2016), the specific allocation method was not recorded explicitly, however the author stated that the pharmacy staff held the randomisation key, and all personnel involved in the recording of date were blinded to the treatment given. The second study (Gruen et al., 2021a) allocated cats on order of entry to the study, but the treatment was not administered in the presence of the owner or data recording personnel. In the third study (Gruen et al., 2021b), randomisation was performed using an electronic data system, which allocated cats to the treatment or placebo groups and assigned them a code which was not broken until statistical analysis. The dispenser knew the code, but not the treatment assigned to each code.

In addition, alongside veterinary assessment, all three of the studies used subjective measures (owner-completed questionnaires) to quantify the perceived clinical effects of frunevetmab. While subjective measurement is more likely to be subject to bias, it is important to also recognise the effects of being examined in a veterinary clinic on the stress-levels and behaviour of cats, and is therefore not a good way to assess chronic pain in cats (Monteiro & Steagall, 2019). In these studies, it was appropriate that the cats were monitored at home using owner assessment (FMPI and CSOM) as outlined by the author in a previous study (Gruen et al., 2015). These scales have also been used but not yet validated in other studies (Lascelles et al., 2007; and Benito et al., 2013). Objective measurement in the form of activity monitors in the two pilot studies was used to try and mitigate this, however in one of the pilot studies (Gruen et al., 2021a) a decrease in activity across both study groups was measured. In this case the author suspected that the baseline had been falsely elevated as the cats were wearing unfamiliar collars with the activity trackers attached, however this was not observed in the first pilot study (Gruen et al., 2016).

A strong placebo-effect has been documented in similar studies involving the assessment of dogs with chronic pain and lameness caused by OA (Conzemius & Evans, 2012). This was also seen in these studies with cats, particularly in the later stages of the longest study (Gruen et al., 2021b). Despite this, there was still a statistical difference between the treatment group and the placebo-group in the majority of time points throughout the studies, which indicates an improved treatment effect in the treatment group over the placebo group. When objective and subjective assessments were combined, the evidence showed that there was an overall improvement in clinical signs associated with degenerative joint disease (DJD) in the cats treated with frunevetmab.

Overall, the strength of the evidence was deemed to be moderate. Chronic pain is a complex subject to study due to the multi-dimensional effects that it has on the patient. These effects can include physical, and behavioural effects. The exclusion criteria for all three studies also means that the cats studied are unlikely to be representative of patients in real clinical situations. Also, despite the strong study designs, all three trials were carried out by the same research group and funded by the manufacturing drug company with their active involvement in the studies.

Therefore, the clinical bottom line is that there is some indication that fully felinised anti nerve growth factor (anti-NGF) monoclonal antibodies are effective at reducing clinical signs in cats with DJD when compared to no treatment.

Further clinical trials, with larger sample sizes, carried out across a larger number of veterinary practices for a longer duration, are warranted to investigate the clinical effects of frunevetmab on OA. It would also be useful in addition to observe the benefits of frunevetmab and compare the treatment effects with an NSAID such as meloxicam to reflect a common patient demographic.

In clinical practice, the suggestion of administering a monthly injection of frunevetmab can be discussed with owners on diagnosis or progression of OA.

## Methodology

**Search strategy:** 

Databases searched and dates covered:	CAB Abstracts (2010–2023) PubMed (2010–2023)
Search strategy:	CAB Abstracts: (Feline or Cat*) and (frunevetmab or NV-02 or solensia or monoclonal antibod*) and (osteoarthritis or arthritis or OA or DJD or degenerative joint disease) PubMed: (Feline or Cat*) and (frunevetmab or NV-02 or solensia or monoclonal antibod*) and (osteoarthritis or arthritis or OA or DJD or degenerative joint disease)
Dates searches performed:	23 Mar 2023

**Exclusion / inclusion criteria:** 

Exclusion:	Not specifically studying cats to answer the PICO question. Papers not written in English. Systematic reviews and book chapters.
Inclusion:	Specifically studying cats to answer the PICO question. Written in English. Controlled clinical trial studies and pilot studies.

**Search outcome:** 

Database	Number of results	Excluded – Not specific to cats or did not answer the PICO question	Excluded – Narrative literature reviews, articles and book pages	Total relevant papers
CAB Abstracts	18	8	7	3
PubMed	47	42	2	3
Total relevant papers when duplicates removed				3

## ORCiD

Lydia Seeley: https://orcid.org/0000-0002-1437-2919

## Conflict of interest

The author declares no conflicts of interest.

## Disclaimer

Knowledge Summaries are a peer-reviewed article type which aims to answer a clinical question based on the best available current evidence. It does not override the responsibility of the practitioner. Informed decisions should be made by considering such factors as individual clinical expertise and judgement along with patient’s circumstances and owners’ values. Knowledge Summaries are a resource to help inform and any opinions expressed within the Knowledge Summaries are the author’s own and do not necessarily reflect the view of the RCVS Knowledge. Authors are responsible for the accuracy of the content. While the Editor and Publisher believe that all content herein are in accord with current recommendations and practice at the time of publication, they accept no legal responsibility for any errors or omissions, and make no warranty, express or implied, with respect to material contained within. For further information please refer to our Terms of Use.
